# Dual and poly-use of novel and conventional nicotine and tobacco product use in Europe: Challenges for population health, regulatory policies, and the ways ahead

**DOI:** 10.3389/fpubh.2023.1093771

**Published:** 2023-02-15

**Authors:** Daniel Tzu-Hsuan Chen

**Affiliations:** ^1^Primary Care Epidemiology, Nuffield Department of Primary Care Health Sciences, University of Oxford, Oxford, United Kingdom; ^2^Public Health Policy Evaluation Unit, School of Public Health, Imperial College London, London, United Kingdom

**Keywords:** poly-product use, smoking, dual use, tobacco control, e-cigarettes, heated tobacco products (HTPs)

## Introduction

**Figure 1:**
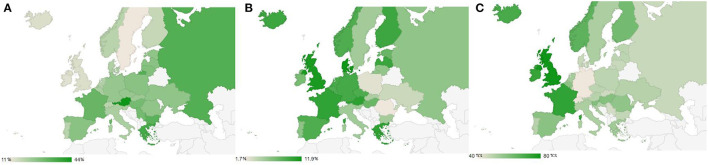
The geographical pattern of cigarette smoking **(A)**, poly-product use **(B)**, and tobacco control scale score **(C)** in Europe. **(A, B)** Prevalence in %. **(C)** The Tobacco Control Scale (TCS) score of total maximum 100 (4). Data source: **(A)** WHO report on the global tobacco epidemic, 2013 (5). **(B)** Chen et al. (7). **(C)** The Tobacco Control Scale 2019 in Europe (4). **(A, B)** Reported prevalence data between 2010 and 2012 for comparability purposes. **(C)** Reported latest TCS score available in 2019 to reflect tobacco control status in Europe, most recent policy improvements may not yet be captured.

Europe has one of the world's highest proportions of premature mortality caused by smoking and tobacco use, with over 700,000 deaths annually across countries in the European Union (EU) (1). Despite significant progress in reducing tobacco use in recent years, tobacco use remains prevalent in the EU, with 26% of the general population and 29% of youths aged 15–24 in this region being current smokers or tobacco users of any type (1, 2). Smoking prevalence varies significantly across European countries ranging from 7% in Sweden to 44% in Greece in 2021 (3). Generally, since the past decade, the prevalence is highest in Central and Eastern European countries as opposed to Western and Nordic countries (Figure 1A).

While the consumption of conventional products such as cigarettes is slowly decreasing, novel nicotine and tobacco products such as e-cigarettes (vaping products) and heated tobacco products (HTPs) are gaining popularity in the European region since 2017, especially among the younger populations (3) and in the countries where the prevalence of conventional tobacco products is high (6). The proliferation of novel or alternative products in the market presents an opportunity for conventional smokers to use these products in combination with conventional ones, leading to dual or poly-product use (7). The 2020 Eurobarometer survey reports a great proportion of current “dual users” of e-cigarette users (59%) and heated tobacco product users (79%), using these products in addition to their current use of conventional products (6).

A recent review showed that the highest prevalence of poly-use (consuming two or more tobacco products concurrently) was mainly observed in Western and Northern Europe and lower in Central and Eastern Europe (7) (Figure 1B). This geographic pattern is generally in contrast to the pattern of smoking. This may suggest that alternative or novel products are taking over the market and being more promoted in regions where conventional products such as cigarettes was more regulated.

The diversity of emerging novel products, their design, and their characteristics make it challenging for countries to regulate and monitor their use at the population level (8). Moreover, growing evidence suggests that dual and poly-product users may be faced with increased health risks and nicotine dependency compared to those using a single tobacco product (9, 10). In the face of such challenges, reinforcing regulations on novel products and tackling dual and poly-product use in Europe should be considered a priority in Europe as part of the vision to achieve tobacco-free generation by 2040 (11).

## Health effects of novel products and risks of dual and poly-product use

People have become more aware of the risks of conventional products such as cigarettes as regulations have been established to limit their use. The tobacco industry is using new marketing strategies to promote novel nicotine and tobacco products that claim to be “harm-reducing” and “safer” and can be used as effective cessation aids (12, 13). However, there is currently insufficient evidence to conclude that any of these novel and emerging products are less harmful than conventional cigarettes. The industry's reduced-risk claim has yet to be fully supported by independent scientific evidence (14, 15).

Recently, there have been studies on the potential role of e-cigarettes. Some evidence suggests that they might help individual smokers quit smoking in clinical settings (16, 17); however, they are not risk-free. Evidence has shown that novel products such as e-cigarettes and HTPs typically contain high levels of nicotine and other toxic substances that can impact human health, causing respiratory illnesses and circulatory disorders and facilitating the development of cancer (14, 15, 18). The World Health Organization (WHO) continues to advise caution on using these products and does not suggest using these devices as a nicotine replacement treatment for current smokers (19).

Evidence on the long-term health effects and potential benefits of novel products is still inconclusive (20). Besides, there are concerns that these products could act as a gateway for non-smokers or the younger population to nicotine addiction (3, 21) or as avenues that lead current smokers to dual and poly-use of these products with conventional products (7, 12). Evidence suggests that dual and poly-use of multiple products can be more addictive and detrimental to health than using a single product alone, as they are exposed to higher levels of nicotine and harmful substances (9, 10).

Given the high prevalence of current dual/poly tobacco use in the European region (6) and the growing market for novel products (12), this is especially concerning at the population level. Without well-enforced regulatory policies, non-smokers could be at risk of taking up tobacco products; smokers may extend their current use status instead of quitting or becoming dual/poly-users concurrently using these products in addition to smoking (22, 23). These could potentially add to the burden of nicotine and tobacco-related diseases, and harm the population.

## Dual and poly-product use and the COVID-19 pandemic

The COVID-19 pandemic has led to unprecedented changes in daily life and people's health behaviors (24). Stress and anxiety during the pandemic may have contributed to an increase in tobacco use or for quitters to relapse (24). Lockdown, quarantine, and changing lifestyles may also have influenced mental health and wellbeing, leading to changes in smoking and health behaviors (24, 25).

With accumulating evidence establishing an association between smoking and a greater risk of COVID-19 disease progression and mortality (26–28), it is plausible that dual tobacco and tobacco use might suffer elevated health risks from the use of multiple products and increase their susceptibility to infection, resulting in a worse prognosis of the virus.

A national representative study in the UK revealed that dual and poly-tobacco uses of novel and conventional tobacco products were associated with 2-fold increased risks of reporting COVID-19 infection compared to non-smokers (29). Furthermore, international studies also highlight dual/poly tobacco users' higher risks of having COVID-19 symptoms and noncompliance with protective behaviors such as social distancing (29, 30). This might reflect social circumstances such as social networking, device sharing, and excessive hand-to-mouth movements related to product use, as these might potentially increase with the use of multiple tobacco products (31).

## Regulating novel products and tackling dual and poly-product use

The implementation of tobacco control policies varies significantly across European countries (Figure 1C). While some core WHO Framework Convention on Tobacco Control (FCTC) measures have been highly implemented in many Western and Northern European countries, some Eastern countries lag with a lower regulatory level. However, the geographic pattern observed in Figure 1C across the European country is generally similar to the patterns of poly-product use seen in Figure 1B. This might imply that effective regulatory policies in countries with stricter regulatory environments in reducing the consumption of novel, non-cigarette tobacco products remain challenging (32), and dual and poly-use of multiple products are more commonly observed (6). This may also reflect the regulatory discrepancies between novel non-cigarette products, which are less regulated and gaining popularity in Europe, and conventional cigarettes typically more strictly controlled in these nations.

To tackle this issue, the European Commission, the Parliament, and the European Council should take the lead in reinforcing national and cross-national tobacco control policies in the European region and beyond. Without expanding regulations to encompass all novel products and a concerted effort to minimize cross-border discrepancies among nations, it may be challenging for existing tobacco control strategies to stay applicable to these products in the face of the shifting landscape of users' behavior of novel products in Europe (6). In accordance with the WHO FCTC, key legislative acts such as the European Union (EU) Tobacco Products Directive (TPD), the Tobacco Tax Directive (TTD), and the Tobacco Advertisement Directive (TAD) should warrant further development and revisions (33). These should include the regulation of novel and emerging products regarding product characteristics/presentation, price/taxation, and advertising/promotion of all related products to reduce overall and dual and poly-product use.

Effective implementation of crucial policies in line with the FCTC MPOWER measures (34) can help address the challenges related to novel nicotine and tobacco products and the growing public health issues caused by dual- and poly-use of tobacco products. This is especially important for nations where the variety of products is expanding. This is necessary to prevent a new generation of users from starting, transitioning, or switching to dual or poly-user status.

- Monitoring tobacco use: There is a need for Europe-wide monitoring data on the use of novel tobacco and nicotine products. Questions regarding single, dual, and poly use of these products should be monitored in surveys consistently and regularly for comparability and representability.
- Protect people from tobacco smoke: To discourage dual and poly use, it is essential to regulate novel products, at least in settings where cigarette smoking is already regulated and where regulation on e-cigarettes and HTPs is still limited in Europe. Extending smoking bans beyond indoors, such as outdoor areas, beaches, and parks.
- Offer help to quit: Cessation guidelines and strategies for quitting novel products should be developed in parallel with conventional tobacco dependencies based on scientific evidence and evidence-based practices to avoid transition and switching between products.
- Warning about the dangers: Stricter packaging and labeling restrictions should apply to e-cigarettes and HTPs to maintain the effectiveness of the TPD against emerging products in the market. Current regulations should be extended further by requiring plain packaging.
- Enforce tobacco advertising, promotion, and sponsorship bans: The Tobacco Advertising Directive has to be updated as tobacco industry marketing to young people through social media is currently not adequately addressed. Advertising and promotion at the point of sale should be restricted to children and adolescents.
- Raise taxes: Taxes should be applied to novel products, in line with national standards, to prevent uptake and transition between products that lead to dual/poly-use. Minimum taxation should be set in member states to prevent price differences that lead to cross-border flows of cigarettes and other product use.

## Discussion

The use of novel products mirrors the use of conventional cigarettes (35). This can contribute to the initiation of cigarette use for youth and non-smokers, and it may encourage current smokers to continue smoking rather than quit or become dual or poly-users of these products (22, 23). These factors could potentially add risks to COVID-19 infection and to the population-level burden of non-communicable diseases (36).

The WHO warned that the growing market for these novel products has threatened existing tobacco control policies and poses a serious barrier to implementing the WHO FCTC (35). This is especially relevant for developing countries in Southeast Asia, the Western Pacific, and the Eastern Mediterranean Regions, where smoking and dual and poly-tobacco use are generally more prevalent than in European countries (7, 37, 38).

As the lines between different types of tobacco and nicotine products are becoming less precise, the tobacco industry can exploit regulatory gaps in marketing and promoting novel products. On the consumer end, without comprehensive policies around these products, current smokers might also take advantage of the regulatory loopholes to switch or transition to other novels, non-cigarette products to circumvent current smoking prohibitions (e.g., smoke-free laws) in certain situations (32). These possibilities could lead to greater dual and poly-use of nicotine and tobacco products, making it more challenging for effective tobacco control regulations and increasing disease burden (7).

Implementing comprehensive, evidence-based, and effectively enforced population-level policies remains a worldwide tobacco control priority. In the face of the expanding market of novel and emerging products, it is critical to implement actions to strengthen synergies and cross-border regulations emphasizing tobacco control to safeguard population-level health, reduce overall product use, and increase cessation.

## Author contributions

DT-HC is the sole contributor to the conceptualization, methodology, analysis, and manuscript preparation of this article. The author contributed to the article have agreed to the published version.
